# Genetic evidence of multiple loci in dystocia - difficult labour

**DOI:** 10.1186/1471-2350-11-105

**Published:** 2010-06-30

**Authors:** Michael Algovik, Katja Kivinen, Hanna Peterson, Magnus Westgren, Juha Kere

**Affiliations:** 1 Department of Clinical Science, Intervention and Technology, Karolinska Institutet, Stockholm, Sweden and Department of Obstetrics and Gynaecology, Västervik Community Hospital, Västervik, Sweden; 2 Wellcome Trust Sanger Institute, Wellcome Trust Genome Campus, Hinxton, Cambridge CB10 1SA, UK; 3 Department of Biosciences and Nutrition, Karolinska Institutet, Stockholm, Sweden

## Abstract

**Background:**

Dystocia, difficult labour, is a common but also complex problem during childbirth. It can be attributed to either weak contractions of the uterus, a large infant, reduced capacity of the pelvis or combinations of these. Previous studies have indicated that there is a genetic component in the susceptibility of experiencing dystocia. The purpose of this study was to identify susceptibility genes in dystocia.

**Methods:**

A total of 104 women in 47 families were included where at least two sisters had undergone caesarean section at a gestational length of 286 days or more at their first delivery. Study of medical records and a telephone interview was performed to identify subjects with dystocia. Whole-genome scanning using Affymetrix genotyping-arrays and non-parametric linkage (NPL) analysis was made in 39 women exhibiting the phenotype of dystocia from 19 families. In 68 women re-sequencing was performed of candidate genes showing suggestive linkage: oxytocin (*OXT*) on chromosome 20 and oxytocin-receptor (*OXTR*) on chromosome 3.

**Results:**

We found a trend towards linkage with suggestive NPL-score (3.15) on chromosome 12p12. Suggestive linkage peaks were observed on chromosomes 3, 4, 6, 10, 20. Re-sequencing of OXT and OXTR did not reveal any causal variants.

**Conclusions:**

Dystocia is likely to have a genetic component with variations in multiple genes affecting the patient outcome. We found 6 loci that could be re-evaluated in larger patient cohorts.

## Background

Dystocia, defined as prolonged and difficult labour, is a common obstetric problem affecting 6-8% of all deliveries [1]. It is a major global health problem due to the increased risk of intrauterine asphyxia of the fetus, operative delivery and subsequently increased risk of both fetal and maternal morbidity such as haemorrhage, infections, pelvic floor trauma, subsequent placenta accreta and neurological disabilities [2-5]. Dystocia followed by instrumental delivery or caesarean section might also be major psychological trauma leading to increased levels of anxiety that by some authors even has been classified as post traumatic stress disorder [6,7].

Parturition is regulated by many factors and the mechanisms regulating labour and the expulsion of the child are incompletely understood [8]. Animal studies have shown that knock-out mice models of genes regulating parturition exhibit dystocia [9,10], but this has not yet been shown in humans [11]. A strong genetic influence has been shown in other obstetric conditions such as preeclampsia, low birth weight, and abnormal gestational length [12,13]. Studies have also detected an increased risk for dystocia in a woman with affected mother or sister [14,15]. We have previously estimated the heritability of dystocia to be 28% [16] and this finding has encouraged us to perform a genome-wide scan in a material consisting of affected sib pairs to further assess the genetic basis for dystocia.

## Methods

### Study population

We used the Swedish Medical Birth Registry [17] that covers almost all births in Sweden to identify women who had given their first birth with caesarean section between 1982 and 1997 at a gestational length of more than 286 days. The reason for this was to have a well defined end point and to increase the chance of finding subjects with dystocia since this condition is more frequent in primiparas and in prolonged gestation and a majority of operative deliveries after 41 weeks are emergency caesarean sections. Using the Swedish Multigeneration Registry [18] we managed to connect 75 sister pairs where both siblings fulfilled the above-mentioned criteria and subsequently contacted them for inclusion in the study. One of the investigators (M. Algovik) performed a structured telephone interview with all women to verify their eligibility to participate in the study, and to investigate whether their mothers had experienced problems giving birth or if there were any other close relatives with such problems. The telephone interview also included questions about the women's subsequent obstetric history and their current health status. To be able to make a simple evaluation of the women's overall experience of the delivery they were also asked to grade it on a five-step scale from very bad to very good. All participants gave their written consent to participate in the study and permission to review the medical records from their first delivery. The diagnosis was confirmed by study of the medical charts of the delivery including partogram. Women with breech presentation, contracted pelvis (sum of the pelvic diameters < 29.5 cm) or a child weighing more than 5000 g were not included in the genetic analysis (NPL). Similarly, multiple pregnancies and families where one or more members refused to participate were excluded.

Following the telephone interviews and the study of the medical records we concluded that the phenotype is heterogeneous since not all of the women had dystocia as an indication for their caesarean section. Given that the primary focus of the study was uterine dysfunction rather than other reasons for dystocia and to enable us to pick the affected sib pairs with the most uniform phenotype for genetic analysis we divided the diagnosis of dystocia into four subgroups: certain dystocia, likely dystocia, unlikely dystocia and no dystocia. These subgroups are described below:

1. Certain dystocia: Definite diagnosis (see Table 1), all subsequent deliveries by caesarean section without other indications.

2. Likely dystocia: Induction of labour, long delivery time (> 20 h), all subsequent deliveries by caesarean section or instrumental delivery and no other indications.

3. Unlikely dystocia: No induction of labour, short delivery time (< 20 h), other indication for caesarean i.e. relative disproportion, child ≥ 5000 g, subsequent deliveries without caesarean section or instrumental delivery.

4. No dystocia: Absolute disproportion, breech presentation, caesarean section before start of labour, delivery misclassified as caesarean but was actually vaginal.

**Table 1:** Diagnose codes

Diagnose code	Text	ICD revision	Year
657.0, 657.1	Prolonged labour, primary and secondary	ICD-8	1973-1986
661A-C, 662A-C	Primary and secondary dystocia	ICD-9	1987-1996
O62.0-1, O63.0-1	Primary and secondary dystocia	ICD-10	1996-

Additional sisters and cousins were invited to participate in families where several women were identified with delivery-related problems. Of the initial 150 women, 56 were excluded and 10 new cases added making the study population a total of 104. Of these 104 women, 83 provided a blood sample for the genetic analysis. Only families with women in group 1 and 2 were used in the genetic analysis with non-parametric linkage (NPL). See Table 2 for details regarding the study population. The study protocol was approved by the institutional review board at Karolinska University Hospital, Huddinge, Sweden.

**Table 2:** Description of sample set

	Number of women	Details
Extraction MBR	150	Sister pairs where both had undergone caesarean section at a gestational length of more than 286 days
Excluded directly	2	One deceased and one emigrated
Excluded after interview	54	One or both sisters refused to participate
Added	10	Additional sisters or cousins

**Total**	**104**	
Blood samples collected	83	
Blood samples analyzed, Affymetrix	39	These women were included in the genetic analysis (NPL)
Blood samples analyzed, re-sequencing	68	These women were included in the re-sequencing of *OXT *and *OXTR*

### Genotyping and genetic analysis

Single nucleotide polymorphism (SNP) genotyping was performed for 18 affected sib-pairs and one family with three affected siblings using the Affymetrix GeneChip^® ^Mapping 10 K 2.0 array containing approximately 10,000 SNP markers following the manufacturer's instructions (Affymetrix, Santa Clara, CA, USA).

MERLIN [19] was used to calculate the Whittemore and Halpern non-parametric linkage (NPL) scores [20]. NPL is a way of measuring how often certain genetic areas are inherited together. Marker positions on DECODE genetic map were retrieved from Affymetrix using NetAffx™ [21], and allele frequencies were estimated using Caucasian allele frequencies provided by Affymetrix. We had low power to detect genotyping errors due to study design, but MERLIN flagged 124 genotypes as unlikely and these were removed from linkage analysis. All individuals were analyzed as affected. NPL scores were calculated for all families, and graphs were created using GNUPLOT. In the graphs, x-axis represents the DECODE genetic map locus, and y-axis represents the NPL score. Genome-wide and chromosome-wide significance of NPL-scores was estimated by simulating data 1,000 times with Merlin and extracting the highest NPL-score from each simulation. Physical positions of peak regions were verified manually against the NCBI dbSNP build 126, which gives chromosomal coordinates for human genome build 36.

### Re-sequencing

Genomic DNA from 68 affected individuals with dystocia and a historic reference material consisting of 107 healthy women without adverse obstetric history who had given a written consent to participate in studies on complications of pregnancy were used to sequence oxytocin (*OXT*) and oxytocin receptor (*OXTR*) genes. Cases were not matched with the reference group, but were used to establish an estimate of the population frequency of potential mutations. Exons including 100 bp flanking sequence on both sides and 1 kb upstream of the first exon were amplified using polymerase chain reaction (PCR). Purified PCR products were sequenced using DYEnamic ET dye terminator kit following manufacturer's instructions (Amersham Biosciences, Buckinghamshire, UK) and electrophoresed using a MegaBACE 1000 instrument (Amersham Biosciences, Uppsala, Sweden). Sequence analysis was performed using the MegaBACE Sequence Analyser 3.0 software (Amersham Biosciences) and Staden package computer programs [22].

## Results

### Characteristics of the study population

We identified 76 women (73.1%) exhibiting a phenotype of certain or likely dystocia. Since many of the subjects were post-term, 53 (51.0%) of them had undergone induction of labour. In 16 of the 47 families (34.0%), the mother had had dystocia or some other obstetric problem such as instrumental or breech delivery. Thirty-eight (36.5%) women said that the delivery had been a bad or very bad experience and 22 (21.2%) said that their first delivery had negatively influenced the number of children they had born. Clinical characteristics have been collected in Table 3 for all interviewed women, Table 4 for the affected sib pairs included in the genetic analysis (NPL) and Table 5 for the 68 women that were used for the re-sequencing of *OXT *and *OXTR*. Since additional female relatives were included after the initial selection to create complete pedigrees some of these values differ from the original selection criteria.

**Table 3:** Clinical characteristics of all interviewed women (n = 104)

Characteristic	Mean	Range	SD
Age	30.2	19.2-43,1	5.0
BMI	27.4	19.1-39.5	3.7
Gestational length	41 + 6	38 + 0 - 43 + 4	
Time from start of delivery to parturition (h)	26:33	4:25 - 96:00	
Birth weight (g)	3824	2360 - 5260	568

**Table 4:** Clinical characteristics for affected sib pairs included in the genetic analysis (NPL) (n = 39)

Characteristic	Mean	Range	SD
Age	32.3	21.9-43.1	4.8
BMI	27.1	20.3-39.5	3.8
Gestational length	41 + 6	40 + 6- 43 + 1	
Time from start of delivery to parturition (h)	28:01	6:00 - 96:00	
Birth weight (g)	3928	2750 - 4850	454

**Table 5:** Clinical characteristics of the women included in the re-sequencing of OXT and OXTR (n = 68)

Characteristic	Mean	Range	SD
Age	31.0	20.7-43.1	5.0
BMI	27.5	19.1-39.5	3.9
Gestational length	41 + 6	38 + 5- 43 + 3	
Birth weight (g)	3886	2750 - 4850	477

### Genetic analysis, non-parametric linkage (NPL)

According to simulations performed with Merlin, significant P-value (identified by an NPL-score reached in 5% of simulations) would have corresponded to an NPL-score of 3.64, while suggestive P-value (identified by an NPL-score reached at least once per simulation) would have corresponded to and NPL-score of 1.98. Our best linkage peak was located on chromosome 12p12 (NPL score 3.15), and other peaks with suggestive linkage were found on chromosomes 3, 4, 6 and 20. Linkage results are summarized in Table 6. The division into the subgroups with certain, likely, unlikely and no dystocia did not increase the significance of genetic analysis. Similarly, division of data into subgroups according to geographical region did not produce significant change to analysis results. Peak regions contained several apoptosis-related factors, calcium-calmodulin dependent kinases, and phospholipase c-like genes, but we were most interested in oxytocin on chromosome 20, oxytocin receptor on 3, endothelin converting enzyme on 3 and endothelin receptor type A on 4. We did not succeed in identifying any dystocia candidate genes in the linkage peak regions on chromosomes 6, 10 and 12.

**Table 6:** Linkage results

Chr	Marker range	Physical range	NPL score	Genomic P
20	rs2013961 - rs674110	2 - 4 Mbp	2.23 (3.7 Mbp)	0.97
12	rs1405608 - rs722918	20 - 62 Mbp	3.15 (51 Mbp)	0.21
10	rs726176 - rs1409317	97 - 121 Mbp	2.92 (115 Mbp)	0.37
6	rs1979541 - rs979515	159 - 165 Mbp	2.84 (164 Mbp)	0.43
4	rs723794 - rs1464452	147 - 157 Mbp	3.03 (154 Mbp)	0.28
3	rs1508722 - rs2358693,	7 - 22 Mbp,	2.43 (13 Mbp),	0.87
	rs725318 - rs763342	179 - 189 Mbp	2.55 (185 Mbp)	0.74

### Re-sequencing of *OXT *and *OXTR*

Initially, we sequenced all *OXT *and *OXTR *exons including splice sites and putative promoter regions in five individuals with dystocia and one control to detect common variations. One known polymorphism was detected within the *OXT *locus, but did not differ in allele frequency from that expected from dbSNP data. We were unable to sequence exon 2 due to high (> 70%) GC content. Sixteen variations were identified within the *OXTR *locus, of which four were novel and the remaining 12 were included in dbSNP with rs numbers. Eleven *OXTR *polymorphisms were selected for sequencing in 68 dystocia cases (Table 5) to assess their allele frequencies in comparison with a historic reference group consisting of 107 women with characteristics described in Table 7. *OXTR *polymorphisms are summarized in Table 8. All detected polymorphisms, including the three successfully genotyped newly detected ones (rsOXTR_01, 02 and 04), were observed to have population frequencies above 1%, and without even suggestive evidence for association, making it unlikely that they would be involved in risk of dystocia.

**Table 7:** Clinical characteristics of the reference group (n = 107)

Characteristic	Mean	Range	SD
Age	28.4	17.0-43.0	4.7
BMI	22.7	17.2-34.5	3.7
Gestational length	39 + 4	37 + 0-42 + 0	
Birth weight (g)	3540	2560-4540	385

**Table 8:** Sequencing results for oxytocin receptor gene (OXTR). rsOXTR_01-04 are novel polymorphisms detected in this study.

Marker name	Location	Alleles	MAF cases	MAF controls	Chi squarea	P value
rs2268498	Upstream of exon 1	T/C	0.48 (63)	0.40 (99)	1.94	0.16
rs2268497	Upstream of exon 1	A/G	0.47 (65)	0.39 (103)	1.59	0.21
rsOXTR_01	Upstream of exon 1	C/T	0.023 (66)	0.014 (104)	0	1
rs1465386	Upstream of exon 1	G/T	0.095 (68)	0.025 (20)	1.25	0.26
rs3806675	Upstream of exon 1	G/A	0.40 (67)	0.40 (107)	0	1
rsOXTR_02	Upstream of exon 1	G/C	0.05 (62)	0.06 (17)	0	1
rs2301260	Exon 1	G/A	0.11 (66)	0.065 (78)	1.55	0.21
rs968389	Exon 1	A/G	0.40 (63)	0.39 (82)	0.02	0.88
rsOXTR_03	Intron 1	C/T	NA	NA	NA	NA
rs237915	Intron 1	T/C	0.27 (62)	0.37 (20)	1.03	0.31
rs237913	Intron 2	C/A	0.27 (62)	0.37 (20)	1.03	0.31
rsOXTR_04	Exon 3	G/A	0.15 (63)	0.15 (20)	0	1
rs2228485	Exon 3	G/A	NA	NA	NA	NA
rs4686302	Exon 3	C/T	NA	NA	NA	NA
rs237902	Exon 3	G/A	NA	NA	NA	NA
rs1042778	3'UTR	G/T	NA	NA	NA	NA

^a ^Yates corrected Chi square

## Discussion

According to our knowledge, this is the first paper assessing the genetic origin of dystocia through non-parametric linkage analysis. There is strong suggestive evidence of linkage at chromosome 12p12 and we found several possible genes in the areas of interest but none that struck as being solely responsible for this condition. The re-sequencing of oxytocin (*OXT*) and oxytocin receptor (*OXTR*), both of which are obvious candidate genes, did not allow us to identify any potential causal mutations. However, it is possible that we have overseen regulatory variants affecting gene expression, mRNA stability or localization of protein product. It is also possible that we have overlooked true candidate genes on regions showing suggestive linkage on chromosomes 4, 6, 10 and 12. We chose comparison with a healthy historic reference material for the re-sequencing of *OXT *and *OXTR*. Evidently this group differs from our cases regarding age, BMI, gestational length and birth weight but we did not manage to find any mutations. If we actually had found any mutations a comparison with matched controls would of course have been preferable.

The results of the present study may be explained in different ways. One obvious weakness is that the phenotype of dystocia is not strictly defined due to clinical heterogeneity and its diagnosis depends both on patient characteristics and the obstetric experience of the local hospital staff. Thus, the use of the clinical entity dystocia varies according to local preferences and traditions, and institutions are likely to diagnose dystocia in a non-standardized manner. This is well illustrated in Sweden where dystocia prevalence in different delivery wards differs widely between 4 and 33 percent [23]. Since the study design is retrospective and partly based on telephone interviews there is a possibility of recall bias, but the interviews were combined with a thorough review of the medical charts. Traditionally dystocia is attributed to three general causes, the three P's in obstetrics; power, passenger and pelvis. With the present approach excluding fetal-maternal disproportion and maternal overweight we attempted to predominantly study the power (uterine muscle activity) within this phenotype.

Although this may have led to the identification of a more consistent group in regards to poor labour it might also have introduced a certain degree of selection bias. We cannot exclude the possibility that the linkage is in fact to some other condition such as high birth weight or post term pregnancy but since these conditions often are related, the genetics of each might be quite complicated to elucidate.

The present study is an example of a project where data from several medical registers has been combined and where we have been granted permission by the Institutional Review Board to contact the patients directly for obtaining biological samples. This is a quite unique approach and it is notable that this approach was well received by the patients. Only 15 women (10%) declined to participate directly at the telephone interview and no case of offending a woman by this direct approach was encountered. On the contrary, the majority of women were willing to donate blood samples without any compensation. Thus, the direct approach appears feasible and has great potential to increase the scientific value of medical registers.

This study indicates that dystocia is a complex disease that is probably not caused by a single locus disease allele. Knowledge of the genetic architecture of complex diseases is still incomplete and it is possible that the risk for any common disease is dependent on a large number of loci, each with a number of low frequency disease-predisposing alleles [24]. The study population included only a limited sample size making it underpowered for association analysis. Our results thus merely established that the newly detected variants were polymorphisms (population frequency > 1%) and adding more subjects would be necessary to increase the opportunity to perform a well-powered genetic association analysis.

During evolution there is balance between mutation and selection and since dystocia-causing mutations would not be brought on to the next generation in the absence of the possibility of caesarean section, there is most likely genetic heterogeneity responsible for the phenotype. The disease-causing alleles can of course also be propagated by male offspring, but we have so far not studied any possible male phenotype.

Since the occurrence of dystocia is likely to involve allelic variations in several different loci we think that it might be very cumbersome and costly to collect a large enough sample set to reach significance in any single locus with genome-wide linkage analysis. An alternative and complimentary approach would be to focus on extensive re-sequencing of candidate genes in affected individuals and controls to assess genetic variability within candidate loci and identify possible causal variants. We have focused on the maternal genotypes but during pregnancy it is probably of interest to take the genetics of the child into account as well. For example, our study does not assess whether dystocia risk is affected by the properties of the fetus, but this might be worthwhile to pursue further.

## Conclusions

In the overall assessment of a woman giving birth the obstetrician might find the knowledge that epidemiological studies have shown an increased risk of dystocia in a primiparous woman if her mother or sister has experienced the same problem to be useful. Our study indicates that there actually might be a genetic background for dystocia through a strong suggestive evidence of linkage at chromosome 12p12 and also at 5 other loci that might be of interest. We believe that larger studies including patients with a well defined phenotype of dystocia might lead to new insights into the aetiology and physiology of this important condition.

## Competing interests

The authors declare that they have no competing interests.

## Authors' contributions

MA participated in planning the study, collected the material, participated in the genetic and statistical analysis and wrote the main part of the manuscript. HP and KK participated in designing the study, and made the main part of the NPL-analysis and re-sequencing as well as the statistical analysis. JK and MW originated the study, participated in its design and helped in writing the manuscript. All authors read and approved the final manuscript.

## Pre-publication history

The pre-publication history for this paper can be accessed here:

http://www.biomedcentral.com/1471-2350/11/105/prepub

## References

[B1] Cunningham FG Williams Obstetrics 20th Edition 1997 20 Stamford, Appleton & Lange

[B2] Macara LM Murphy KW The contribution of dystocia to the cesarean section rate [see comments] American journal of obstetrics and gynecology 1994 171 1 71 77 7794300 10.1016/s0002-9378(94)70080-x

[B3] Chelmow D Kilpatrick SJ Laros RK Jr Maternal and neonatal outcomes after prolonged latent phase Obstetrics and gynecology 1993 81 4 486 491 8459953

[B4] Saunders NS Paterson CM Wadsworth J Neonatal and maternal morbidity in relation to the length of the second stage of labour British journal of obstetrics and gynaecology 1992 99 5 381 385 1622909 10.1111/j.1471-0528.1992.tb13753.x

[B5] Silver RM Landon MB Rouse DJ Leveno KJ Spong CY Thom EA Moawad AH Caritis SN Harper M Wapner RJ Sorokin Y Miodovnik M Carpenter M Peaceman AM O'Sullivan MJ Sibai B Langer O Thorp JM Ramin SM Mercer BM Maternal morbidity associated with multiple repeat cesarean deliveries Obstetrics and gynecology 2006 107 6 1226 32 16738145 10.1097/01.AOG.0000219750.79480.84

[B6] Nystedt A Hogberg U Lundman B The negative birth experience of prolonged labour: a case-referent study Journal of clinical nursing 2005 14 5 579 586 10.1111/j.1365-2702.2004.01105.x 15840072

[B7] Ryding EL Wijma K Wijma B Psychological impact of emergency cesarean section in comparison with elective cesarean section, instrumental and normal vaginal delivery J Psychosom Obstet Gynaecol 1998 19 3 135 144 10.3109/01674829809025691 9844844

[B8] Dizon-Townson D Ward K The genetics of labor Clinical obstetrics and gynecology 1997 40 3 479 484 10.1097/00003081-199709000-00005 9328727

[B9] Mahendroo MS Cala KM Russell DW 5 alpha-reduced androgens play a key role in murine parturition Molecular endocrinology (Baltimore, Md) 1996 10 4 380 392 10.1210/me.10.4.380 8721983

[B10] Sugimoto Y Yamasaki A Segi E Tsuboi K Aze Y Nishimura T Oida H Yoshida N Tanaka T Katsuyama M Hasumoto K Murata T Hirata M Ushikubi F Negishi M Ichikawa A Narumiya S Failure of parturition in mice lacking the prostaglandin F receptor Science 1997 277 5326 681 683 10.1126/science.277.5326.681 9235889

[B11] Algovik M Lagercrantz J Westgren M Nordenskjold A No mutations found in candidate genes for dystocia Human reproduction (Oxford, England) 1999 14 10 2451 2454 10.1093/humrep/14.10.2451 10527967

[B12] Clausson B Lichtenstein P Cnattingius S Genetic influence on birthweight and gestational length determined by studies in offspring of twins Bjog 2000 107 3 375 381 10740335 10.1111/j.1471-0528.2000.tb13234.x

[B13] Arngrimsson R Bjornsson S Geirsson RT Bjornsson H Walker JJ Snaedal G Genetic and familial predisposition to eclampsia and pre-eclampsia in a defined population British journal of obstetrics and gynaecology 1990 97 9 762 769 2242360 10.1111/j.1471-0528.1990.tb02569.x

[B14] Berg-Lekas ML Hogberg U Winkvist A Familial occurrence of dystocia American journal of obstetrics and gynecology 1998 179 1 117 121 10.1016/S0002-9378(98)70260-1 9704775

[B15] Varner MW Fraser AM Hunter CY Corneli PS Ward RH The intergenerational predisposition to operative delivery Obstetrics and gynecology 1996 87 6 905 911 10.1016/0029-7844(96)00064-6 8649696

[B16] Algovik M Nilsson E Cnattingius S Lichtenstein P Nordenskjold A Westgren M Genetic influence on dystocia Acta obstetricia et gynecologica Scandinavica 2004 83 9 832 837 15315594 10.1111/j.0001-6349.2004.00544.x

[B17] Cnattingius S Ericson A Gunnarskog J Kallen B A quality study of a medical birth registry Scand J Soc Med 1990 18 2 143 148 2367825 10.1177/140349489001800209

[B18] Lunde AS Lundeborg S Lettenstrom GS Thygesen L Huebner J The person-number systems of Sweden, Norway, Denmark, and Israel Vital Health Stat 1980 2 84 1 59 7445446

[B19] Abecasis GR Cherny SS Cookson WO Cardon LR Merlin--rapid analysis of dense genetic maps using sparse gene flow trees Nature genetics 2002 30 1 97 101 10.1038/ng786 11731797

[B20] Whittemore AS Halpern J Probability of gene identity by descent: computation and applications Biometrics 1994 50 1 109 117 10.2307/2533201 8086594

[B21] Liu G Loraine AE Shigeta R Cline M Cheng J Valmeekam V Sun S Kulp D Siani-Rose MA NetAffx: Affymetrix probesets and annotations Nucleic acids research 2003 31 1 82 86 10.1093/nar/gkg121 12519953 PMC165568

[B22] Bonfield JK Smith K Staden R A new DNA sequence assembly program Nucleic acids research 1995 23 24 4992 4999 10.1093/nar/23.24.4992 8559656 PMC307504

[B23] Högberg U Dystocia in Sweden (from Medical Birth Registry) 2004 Personal communication

[B24] Reich DE Lander ES On the allelic spectrum of human disease Trends Genet 2001 17 9 502 510 10.1016/S0168-9525(01)02410-6 11525833

